# Clinical characteristics and outcomes of patients with Herpes Zoster Infection in the context of SARS-CoV-2 infection. A case report and a systematic review

**DOI:** 10.5339/qmj.2022.41

**Published:** 2022-09-01

**Authors:** Pawan Kumar Thada, Fateen Ata, Muhammad Ali, Mohammad Nasser Affas, Jenish Bhandari, Sarosh Sarwar, Bilal Ahmed, Hassan Choudry

**Affiliations:** ^1^Department of Internal Medicine, Faisalabad Medical University, Faisalabad, Pakistan E-mail: docfateenata@gmail.com; E-mail: FAta@hamad.qa; ^2^Department of Internal Medicine, Hamad Medical Corporation, Doha, Qatar; ^3^Department of Internal Medicine, SUNY Upstate Medical University, USA; ^4^Department of Internal Medicine, Fazaia Medical College, Pakistan; ^5^Department of Internal Medicine, Heartlands Hospital, Birmingham; ^6^Department of respiratory medicine, University Hospital of Leicester, UK

**Keywords:** Coronavirus, COVID-19, SARS-CoV-2, Herpes zoster, Varicella zoster virus, vesicular rash, virus reactivation

## Abstract

Introduction: Severe acute respiratory syndrome-coronavirus 2 (SARSCoV2) pandemic has been an unceasing plight with a wide range of clinical presentations. The direct effects of the virus, increased use of medications, and lifestyle changes have contributed to the vulnerability to co-infections. Fungal and bacterial co-infections led to increased morbidity and mortality during the pandemic. Similarly, the surge of skin signs in conjunction with herpes zoster (HZ) manifestations has been reported. In this study, we pooled the data on the clinical characteristics of SARS-CoV-2 patients co-infected with HZ.

Methodology: Electronic databases including PubMed, Scopus, and Google Scholar were extensively searched to identify the relevant studies on HZ infection among the SARS-CoV-2 patients.

Results: A total of 79 patients (from case reports, series, and retrospective studies) were included in the analysis. Fever was the most common constitutional symptom recorded, followed by cough and dyspnea. A systemic rash was reported in 78.5% of cases with mild symptoms of HZ and SARS-CoV-2 in 87% and 76%, respectively. Only 19% of the cases presented during the prodrome period of SARS-CoV-2. HZV polymerase chain reaction (PCR) was positive in 8.9% of the cases, and the remaining were diagnosed clinically. SARS-CoV-2 PCR was reported positive in 65 cases (82.3%). Leukopenia was observed in 7 cases (8.9%) and lymphopenia in 25 (31.6%). All patients recovered through conservative treatment.

Conclusion: SARS-CoV-2 escalated the incidence of HZ reactivation. Most of the patients were seen with older individuals either simultaneously or a few days after the SARS-CoV-2 infection, but a few cases were reported during the asymptomatic prodrome period of SARS-CoV-2

**Registration:** This review is registered in *The International Prospective Register of Systematic Reviews (PROSPERO), National Institute for Health Research (NIHR)* (CRD42022301821) https://www.crd.york.ac.uk/PROSPERO/display_record.php?RecordID = 301821

## Introduction

Coronavirus disease 2019 (COVID-19) caused by the SARS-CoV-2 virus continues to remain a global pandemic with more than 400 million cases reported across the world.^1^ Infection with the SARS-CoV-2 virus presented with a wide spectrum of symptoms, ranging from mild disease to multi-organ severe diseases. Mild SARS-CoV-2 infection mainly presented with symptoms such as flu-like illness (including fever, myalgia, cough, shortness of breath, loss of taste or smell, and diarrhea). Moderate to severe SARS-CoV-2 infection with pulmonary system involvement led to increased shortness of breath, requiring oxygen, cyanosis, altered mental status, confusion, chest pain, and acute respiratory distress syndrome (ARDS) requiring ventilatory support.^2^ There have been reports on a multitude of atypical presentations and associated co-infections with SRAS-COV-2.^3^ Dermatological manifestations such as maculopapular and vesicular rashes, erythema multiforme-like eruptions, pityriasis rosea, vascular rashes, urticarial rashes, chilblain-like lesions, livedoid lesions, petechiae, and purpura lesions are rare.^4,5^

The active infectious phase of the SARS-CoV-2 virus and post-SARS-CoV-2 physical state possibly served as a favorable environment for invasion and reactivation of other infections due to lymphopenia caused by several mechanisms.^6–9^ Multiple cases of reactivation of herpes zoster (HZ) were reported along with COVID-19 disease or during the post-infection phase.^10^ Skin manifestations of COVID-19 and the concurrent infectious etiology including HZ may be mistaken if not diagnosed meticulously. SARS-CoV-2 infection is a potential cause of immunosuppression with multimodal pathophysiology. Wang et al. reported that severe cases of SARS-CoV-2 had a lower level of total lymphocytes, CD4+T cells, CD8+T cells, and B cells.^11^ Secondly, pandemics led to physical and psychological stress. As the COVID-19 pandemic has entered its 3^rd^ year, stress is becoming chronic and leading to immunosuppression.^12^ Moreover, corticosteroids have been widely used throughout the world for the treatment of SARS-CoV-2, which is a distinct cause of immune dysregulation.^13^ The unceasing picture of a pandemic due to evolving new variants of SARS-CoV-2 provides a nidus for several opportunistic co-infections like HZV. Although multiple cases and small case series have reported co-infection of HZ with SARS-CoV-2, the literature is devoid of any large-scale study. Extensive studies on such co-infections are imperative to assess the true burden of the co-infections, with a focus on the etiology and its effects on the pandemic. In this context, our study is one of the first extensive systematic reviews that has pooled and presented SARS-CoV-2 cases co-infected with HZV. We have also highlighted the demographics, clinical characteristics of these patients, disease severity, clinical presentations, and the outcomes of these cases.

## Methods

### Literature review

We conducted a systematic review of all reported cases that discussed HZ infection in the context of COVID-19 across PubMed, Scopus, and Google Scholar from inception until January 4, 2022. We used the following MeSH terms for the search: ((“covid 19”[All Fields] OR “covid 19”[MeSH Terms] OR “covid 19 vaccines”[All Fields] OR “covid 19 vaccines”[MeSH Terms] OR “covid 19 serotherapy”[All Fields] OR “covid 19 serotherapy”[Supplementary Concept] OR “covid 19 nucleic acid testing”[All Fields] OR “covid 19 nucleic acid testing”[MeSH Terms] OR “covid 19 serological testing”[All Fields] OR “covid 19 serological testing”[MeSH Terms] OR “covid 19 testing”[All Fields] OR “covid 19 testing”[MeSH Terms] OR “sars cov 2”[All Fields] OR “sars cov 2”[MeSH Terms] OR “severe acute respiratory syndrome-coronavirus 2”[All Fields] OR “ncov”[All Fields] OR “2019 ncov”[All Fields] OR ((“coronavirus”[MeSH Terms] OR “coronavirus”[All Fields] OR “cov”[All Fields]) AND 2019/11/01:3000/12/31[Date – Publication])) AND “Zoster”[Title/Abstract]) NOT “vaccin*”[Title].

### Study selection

**Figure 1.**
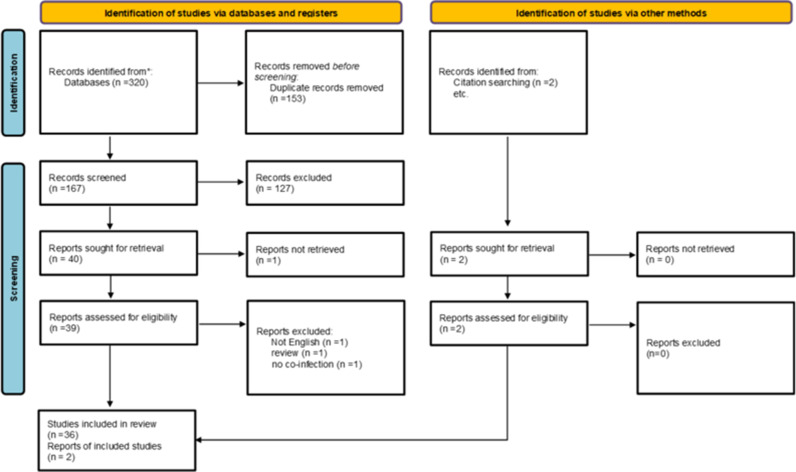
PRISMA flow chart of the study selection process, including the added and excluded studies with details.

After removing the studies that did not meet our inclusion criterion, 38 articles were included in the final review (Figure 1). These articles included case reports, case series, and retrospective studies. We could not identify any prospective study to add to this review. This review was conducted according to the Preferred Reported Items for Systematic Reviews and Meta-analyses (PRISMA) guidelines.^14^ Furthermore, our review has been registered on the International Prospective Register of Systematic Review (PROSPERO; no.: CRD42022301821).

### Data extraction

We developed a standard form for data extraction. Accordingly, we extracted sociodemographic information of the patients, comorbidities, history of prior infections, timelines and management of HZ and COVID-19, details of the steroid use, complications of HZ, and SARS-CoV-2, details of SARS-CoV-2 symptoms, and the duration of hospital stay.

### Inclusion and exclusion criterion

English language studies that reported the emergence of HZ among SARS-CoV-2 patients were added to this review. The exclusion criteria were as follows: studies that did not report original data, for example, any qualitative studies, review articles, and commentaries or conference abstracts; studies on isolated SARS-CoV-2 or HZ infection; studies published in a language other than English; studies wherein a diagnosis of COVID-19 was made without confirmatory polymerase chain reaction (PCR) testing or covid antigen test.

### Quality Assessment

We used the Joanna Briggs Institute critical appraisal tools to assess the quality of the included papers.^15^ One additional retrospective study was assessed using the Methodological index for non-randomized studies (minors).^16^ The selected studies were examined for conformance with the inclusion criterion, sample size, description of study participants, and settings. Two reviewers independently assessed the methodological quality of each paper. As, predominantly, case reports and case series were included in analyses, we did not conduct meta-analyses to maintain accuracy in data reporting.

### Statistical analysis

Descriptive and summary statistics were employed to analyze the data. Means (with standard deviation) and medians (with interquartile ranges), and numbers (with percentages) were reported as deemed appropriate. Shapiro–Wilk test was applied to judge the normality of the data. Correlation analysis was not conducted considering the nature of the study design. All analysis was performed on MS Excel 2016 and SPSS.

## Results

This review involved 79 patients; all of them had COVID-19 and HZ virus co-infection either before, after, or simultaneously with SARS-CoV-2, and 24 of these cases (30.4%) were published as case reports, 39 (49.4%) as case series, and 16 (20.3%) as a single retrospective study.

### Demographics and clinical features

Table 1 summarizes the demographics of the patients. The average age of patients presenting with HZ virus and COVID-19 was 53.97* ± *18.91 years (median age: 56 years), with 46 males (58.2%) and 33 females (41.8%). Fever (n = 39, 49.4%), cough (n = 27, 34.2%), dyspnea (n = 13, 16.5%), myalgias (n = 13, 16.5%), loss of taste and smell (n = 12, 15.2%), and sore throat (n = 10, 12.7%) were the most commonly reported clinical findings, while fatigue (n = 4, 5.1%), diarrhea (n = 4, 5.1%), loss of consciousness (n = 2, 2.5%), nasal congestion (n = 1, 1.3%), chest pain (n = 1, 1.3%), and vomiting (n = 1, 1.3%) were less commonly reported. There have been no reported cases of hemoptysis.

### Epidemiology

In this review, a history of chickenpox and HZ infection was reported in 9 (11.4%) and 5 patients (6.3%), respectively. We noted that 3 patients had immunodeficiency (3.8%) and one had malignancy (1.3%) (a case of diffuse large B cell lymphoma), while recurrent past infections were reported in 4 patients (5.1%). There were no reported cases of HIV/AIDS or dementia.

### Comorbidities

Hypertension was the most reported associated condition (n = 11, 13.9%), followed by diabetes mellitus (n = 4, 5.1%), and chronic kidney disease (n = 3, 3.8%). Chronic lung disease was reported in one patient (1.3%). Table 2 summarizes the comorbidities.

### Clinical manifestations and timelines of HZ infection and COVID-19

Sixty-two patients (78.5%) reported systemic rash and 15 patients (19%) reported HZ infection as the initial symptom requiring hospital admission. Later, all these patients were diagnosed with SARS-CoV-2. The severity of HZ infection was mild (n = 69, 87.3%), moderate (n = 4, 5.1%), or severe (n = 1, 1.3%), while the severity of SARS-CoV-2 was reported to be asymptomatic or mild (n = 60, 76%), moderate (n = 4, 8.9%), or severe with ARDS (n = 8, 10.2%). If these cases, no data was available on the severity of SARS-CoV-2 in 7 cases (8.9%). Moreover, 29 patients developed SARS-CoV-2 symptoms before hospital admission with an average duration of 9.52 days (median: 5 days), and 15 patients presented with HZ earlier than SARS-CoV-2 with an average duration of 2.73 days. In our study, a considerable number of patients developed HZ virus symptoms a few days after SARS-CoV-2 diagnosis. We calculated the average duration separately from the manifestation of SARS-CoV-2 clinical symptoms and the diagnosis of HZ virus rash. The average duration from the first SARS-CoV-2 symptoms to the appearance of HZ virus rash was 15.49 days (median: 7 days, n = 43), while the average duration from SARS-CoV-2 diagnosis to the appearance of HZ virus rash was 14.80 days (median: 6.50 days, n = 4). Data on hospital stay was not available in any of the included papers. Only 3 cases reported hospital stays of 16, 32, and 99 days. Table 3 summarizes the clinical course for symptoms development and diagnosis, whereas Table 4 summarizes the location of HZ rash in the patients.

### Laboratory findings

Seven cases (8.9%) reported positive HZV PCR, one case (1.3%) showed positive HZ virus antigen, and the remaining of them were diagnosed clinically. Although all these cases were diagnosed with SARS-CoV-2, only 65 cases (82.3%) were reported to have available COVID-19 PCR. White blood cells were normal in 10 patients (12.7%), while 7 patients (8.9%) had leukopenia and one patient (1.3%) had leukocytosis. The lymphocyte count was normal in 7 patients (8.9%) and increased in 2 patients (2.5%), while 25 patients (31.6%) had lymphopenia.

### Imaging

Chest X-ray was reported in 9 patients (11.4%), and it was normal in 3 patients (33.3%), while 3 patients (33.3%) reported ground-glass infiltration, 2 patients (22.2%) presented with features of bilateral pneumonia, and one patient (11.1%) had multiple patchy opacities. After a chest CT scan was performed in 22 patients (27.8%), 15 of them (68.2%) presented with ground-glass infiltration, 2 (9%) showed features of bilateral pneumonia, one (4.5%) showed features of Varicella pneumonia indicating pulmonary nodules, and one (4.5%) had discrete interstitial inflammation. CT scan was found to be normal in 2 patients (9%).

### Management

Fifty-nine patients (74.7%) received antiviral therapy for HZV, and the most commonly used agents were acyclovir (n = 42, 53.2%) and valaciclovir (n = 15, 18.98%), followed by famciclovir in 2 patients (2.5%) and valganciclovir in one patient only (1.3%). Only 31 patients reported on the administration route, and the most common route was oral (n = 16, 51.6%), followed by intravenous (n = 13, 41.9%) and topical (n = 2, 6.45%). Eleven patients (13.9%) received steroids, and the most common was prednisolone (n = 7, 8.9%) with doses ranging from 5 mg to 20 mg and dexamethasone (n = 4, 5.1%) with doses of either 4 mg or 8 mg. Four patients (5.1%) received steroids topically and 3 patients (3.8%) received a total of 5 days of steroids. For COVID-19, most commonly used therapies were hydroxychloroquine/chloroquine (n = 14, 17.8%), azithromycin (n = 7, 8.9%), remdesivir (n = 3, 3.8%), tocilizumab (n = 2, 2.5%), and steroids (n = 2, 2.5%). Table 5 summarizes the medications used to treat the HZ virus and COVID-19.

### Outcome and complications

Most common complications attributed to COVID-19 were ARDS (n = 7, 8.9%), need for mechanical ventilation (n = 6, 7.6%), extracorporeal membrane oxygenation (ECMO) (n = 2, 2.5%), and pulmonary embolism (n = 2, 2.5%). HZ complications included neuralgia (including trigeminal neuralgia) (n = 4, 5%) and cases of sensorineural hearing loss, necrotic herpes, and bilateral retinal necrosis (for each: n = 1, 1.3%). Fortunately, mortality was 0% in our review despite 6 patients (7.5%) requiring mechanical ventilation.

### Case presentation

A 36-year-old woman presented with painful blisters for 4 days on the front and lateral side of her right thorax. The pain was severe in intensity, itchy, stabbing, and continuous. The blisters were initially small and few but progressively increased in number and size. Cutaneous examination revealed a group of rashes with watery discharge on the right side of the trunk involving tbl2, tbl3, and tbl4 dermatomes. The diagnosis of HZ was made on clinical grounds. She indicated no relevant comorbidities, intake of immunosuppressives, prior SARS-CoV-2 vaccination, or any autoimmune disorders. However, she had a history of similar rashes on the right half of the loin a year back. She was accordingly treated with oral acyclovir (dose), paracetamol, and an analgesic agent. Her vitals and investigations were within the normal range.

Three days after admission, she developed a cough, fever, shortness of breath, and headache, followed by a loss of smell and taste that started 2 days later. She was accordingly tested for SARS-CoV-2 and the SARS-CoV-2 PCR test turned out to be positive. Considering that the SARS-CoV-2 PCR came positive within 3 days of hospitalization, it was unlikely a nosocomial COVID-19 disease and likely derived from the community. Chest X-ray revealed a bilateral hazy ground-glass appearance. D-dimers (783 ng/mL) and serum ferritin (398 ng/mL) markers were raised. Hb (10.1 mg/dL), leukocytes count (11900/cmm), and platelets count (181000/cmm) were in the normal range, but lymphopenia (10%) was noted. High-resolution computed tomography chest demonstrated well-appreciated multiple bilateral peripherally placed patchy areas of ground-glass haze along with patchy consolidation in the upper and lower lobes with approximately 60–70% lung parenchyma involvement, suggestive of COVID-19 pneumonia. The hospital protocol for SARS-CoV-2 (supportive management) was initiated. The investigations were performed every 3–4 days and as per the requirement. Hb (10.6 mg/dL) and platelets count (195000/cmm) remained in the normal range, but lymphopenia persisted and then worsened (Table 6). Herpes rashes were necrosed and skin mupirocin ointment was applied. Finally, she maintained saturation without O_2_ support. D-dimers (310 ng/mL), serum ferritin (120 ng/mL), C-reactive protein (7 mg/dL), lactate dehydrogenase (420 u/L), and lymphocytes (17%) also improved. She was discharged after 20 days of hospitalization with home medications and precaution guidelines.

## Discussion

This systematic review addresses the correlation between HZ and SARS-CoV-2, with a focus on the clinical characteristics of patients, timelines of the two infections, management, and outcomes of HZ infection and COVID-19. In this paper, we disserted the HZV manifestations during SARS-CoV-2 infection and its possible mechanisms. With respect to SARS-CoV-2, increased mortality has been reported among patients with fungal or bacterial co-infections.^17^ However, the literature lacks the morbidity and mortality burden of SARS-CoV-2 and viral co-infections. Concerns regarding the association of HZ reactivation in SARS-CoV-2-infected patients were raised due to an increased incidence during the pandemic.^18^ Past studies reported an increased incidence of HZ with age, with a peak in the incidence at 60–70 years of age. This was most probably due to an age-related reduction in lymphocytes and immunosenescence.^19^ In our review, the reported cases of concomitant HZ infection and SARS-CoV-2 revealed a similar trend (median age: 56 years). The average global pre-pandemic prevalence of HZ was 3–5/1000 to 10–12/1000 in older and severely immunocompromised individuals.^19,20^ Emerging data indicates an increase in this prevalence.^10^ SARS-CoV-2, the conspicuous immunosuppressant, is probably the cause of increased HZV reactivation during the pandemic.^21^ Notably, a Brazilian study revealed that HZ cases increased by 10.7 cases per million when compared to that during the pre-pandemic era.^22^ HZ demonstrated a strong association with HIV-infected patients, stem cell or solid organ transplantation, and immunosuppressive therapy. HZ virus reactivation was almost double in immunocompromised patients than in healthy individuals, and the rate increased with the severity of immunosuppression.^20^ However, our review found that HZ virus reactivation in COVID-19-associated patients was less likely to have other causes of immunodeficiency. In other words, only 1–3% of the cases were reported without any evidence of HIV infection despite HIV being the most common cause among the general population.^23^

Herpes virus remains in a latent state due to its intracellular spread and neuronal resistance to VZV-induced apoptosis, possibly through downmodulating the expression of surface ligands recognized by natural killer cells, although these receptors were upregulated in various stressful conditions such as SARS-CoV-2.^24–26^ SARS-CoV-2 infection could be the stressor that facilitated the activation of VZV. Cell-mediated immunity is contributed primarily by T cells, which include both CD4+ and CD8+ cells. This mechanism plays a major role in the inactivation of latent HZ in immunocompetent individuals with intact T cell activity. Past studies have shown that >80% of the patients suffered from lymphopenia, and its severity aligns with the intensity of COVID-19 infection.^7,27^ Several cases of herpes virus infections in patients with positive SARS-CoV-2 demonstrated a decrease in the T lymphocyte subpopulations and, specifically, that in CD4+ and CD8+T lymphocytes before the onset of HZ.^28,29^ SARS-CoV-2 reduces CD4^+^T cells more severely than CD8^+^T cells. Analysis by Wang et al. revealed a significant SARS-CoV-2-induced reduction in lymphocytes and NK cells. Moreover, B cells, eosinophils, and monocytes were affected, but less severely, than T lymphocytes.^11^ This event translated into a temporary immunosuppression state of the SARS-CoV-2 infection. Past studies suggested that lower immune status is associated with a more complicated HZ disease course and greater long-term effects.^19,20^ Although the exact lymphocyte count was not available in any of our included papers (available only in 31.6% of all cases), lymphopenia was a prominent feature in a majority of cases. Lymphopenia caused by SARS-CoV-2 provides an opportunity for the reactivation of the HZ virus. This point was reinforced by the fact that, in a majority (81%) of cases, HZ infection was diagnosed simultaneously or after a few days of COVID-19 diagnosis; hence, immunosuppression caused by SARS-CoV-2 led to HZ virus reactivation in these cases. However, the virus can also be reactivated during the prodrome period of SARS-CoV-2 as present in some of our cases. Table 3 summarizes our findings with respect to the duration of symptoms of COVID-19 and HZ infection.

**Figure 2.**
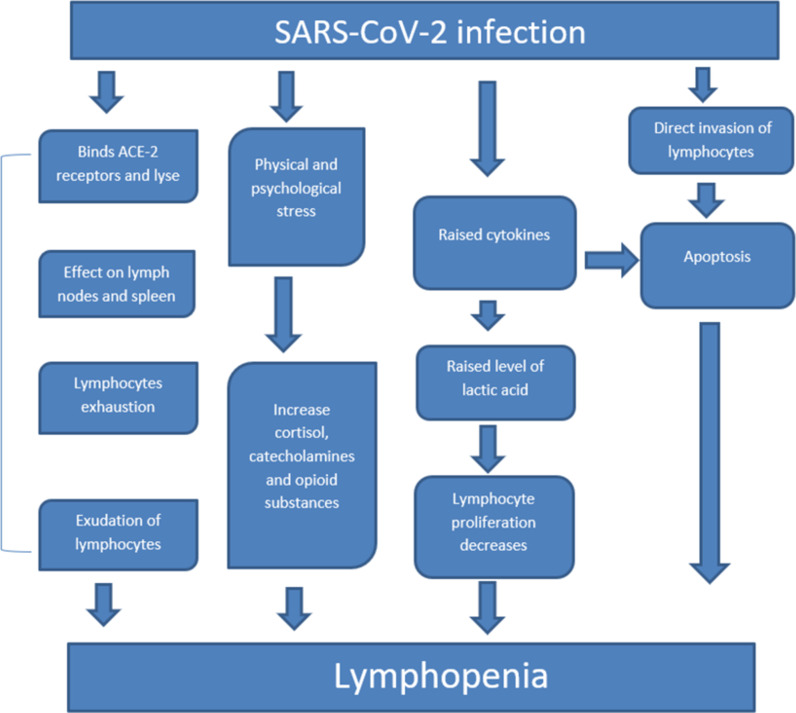
Proposed pathophysiology of SARS-CoV-2-led immunosuppression that might be the potential trigger of co-infections.

SARS-CoV-2-associated lymphopenia has multiple hypotheses of pathophysiology.^30,31^ First, the SARS-CoV-2 virus could directly invade the lymphocytes, induce apoptosis, and lead to impairment of antiviral response. Second, the SARS-CoV-2 virus possesses the ability to lyse the lymphocytes binding angiotensin-converting enzyme 2 (ACE2) receptors on the surface of T lymphocytes. Third, the lymphatic organs such as the lymph nodes and the spleen were affected by SARS-CoV-2. Fourth, elevated levels of cytokines [TNF-a, IL-6] as a result of SARS-CoV-2-led damage-induced apoptosis and raised the lactic acid concentration, which halted the proliferation of lymphocytes and significantly contributed to lymphopenia.^6,7,32^ Moreover, the exudation of circulating lymphocytes into the inflammatory lung tissues may also cause lymphopenia.^11^ Finally, lymphocyte exhaustion following initial hyperactivation also contributed to SARS-CoV-2 infection-induced lymphopenia.^33^ All these findings were different from those of pneumonia caused by other common respiratory viruses, which are usually associated with a normal or elevated lymphocyte count.^34^ Figure 2 summarizes the possible pathophysiology of SARS-CoV-2-led immunosuppression. In some cases, SARS-CoV-2-associated psychological trauma may be a trigger for HZ virus reactivation.^35,36^ Continuous stressful condition is also a nidus for virus reactivation and its progression.^24^ SARS-CoV-2 imposes physical and psychological stress in both infected and non-infected general populations, which increases the risk of shingles.^36^ Studies have demonstrated that quarantine led to exhaustion,^33^ detachment from others, anxiety, irritability, insomnia, poor concentration, indecisiveness, deteriorating work performance, avoidance behavior, anger, fear about self-health or fears of infecting others, loss of usual routine, reduced social and physical contact, frustration, a sense of isolation from the rest of the world, and stigma.^37^ Poor information from public health authorities and insufficient clear guidelines about actions to take are also significant causes of stress.^38^ This anxiety, depression, and stress together increase the levels of cortisol, catecholamines, and certain opioid substances that lead to immunosuppression in several ways, such as lymphocytopenia and hypogammaglobulinemia.^39^

In our review, in some cases, the symptoms of HZ appeared before those of SARS-CoV-2. Elsaie et al.^40^ described 2 cases of COVID-19 who initially presented with HZ lesions.^40^ This case report suggested that HZ can be an indicator for latent SARS-CoV-2 infection. During the current SARS-CoV-2 pandemic, the presentation of HZ in patients with mild upper respiratory tract symptoms served as a signal for latent SARS-CoV-2 infection.^41^

Recalcati 2020 reported, for the first time, the involvement of skin manifestations with the COVID-19 disease. Recently, there have been many reports on patients with features of skin rash after SARS-CoV-2 disease, which ranged from erythematous rash to chickenpox-like/urticarial eruption.^42^ Although SARS-CoV-2-infected patients are reported to have presented with ample skin manifestations resembling the herpes virus, the lesions associated with SARS-CoV-2 disease were less itchy and mostly detected in the trunk and limbs and rarely on the face as compared to the typical localized dermatomal appearance of HZ.^43^ Besides skin manifestations, few papers corroborated the VZV encephalitis, retinal necrosis, and cranial polyneuropathy.^44,45^ Ferreira et al. reported an HZ case, wherein SARS-CoV-2 disease symptoms developed 10 days later.^24^ In this case, SARS-CoV-2 may have fostered a retrograde reactivation of VZV from the nasal cavity, where ophthalmic and maxillary branches of the trigeminal nerve were harbored. Hence, SARS-CoV-2 disease may also entail this rare presentation of HZ as it is unusual for all 3 divisions of the trigeminal nerve to be affected by SARS-CoV-2 infection.^24^ A rare manifestation of SARS-CoV-2 patients with disseminated multi-dermatomal HZ infection could be due to the involvement of CD4 and CD8 T cells in the SARS-CoV-2 infection that allowed the virus to spread across the body as in immunodeficient persons.^46^ Solanki et al. reported a case of disseminated HZ due to temporary immunosuppression caused by SARS-CoV-2.^47^

Steroids are commonly used for the treatment of severe SARS-CoV-2, and they are known to cause immunosuppression.^48^ However, our study hints that the main culprit behind the reactivation of HZ may be immunosuppression caused by SARS-CoV-2 rather than by steroids because only 2.5% of the included SARS-CoV-2 patients used steroids. These results need validation, preferably in prospective study designs to overcome the biases that could have impacted our findings.

Despite the double burden of infections, interestingly, all patients recovered with the traditional antiviral and symptomatic management. The possible reason for this can be mild infection (87.3%) with SARS-CoV-2 in our included patients and a smaller number of comorbidities like cancer, cardiac issues, and liver diseases in the majority of our included patients considering that hypertension is present only in 13.8% and CKD in 3.8% of patients. Only a single patient in our review demonstrated diffuse large B cell lymphoma. Moreover, the length of hospitalization was not significantly increased in SARS-CoV-2 patients who were co-infected with the HZ virus when compared to that in SARS-CoV-2 patients alone. The median duration of hospitalization in our study was 32 days. A systematic review by Rees et al. reported that the median duration of hospitalization ranged from 5 to 29 days in SARS-CoV-2 patients with a median hospital length of stay of 4–53 days in China and 4–21 days outside China.^49^ Our results indicate that, unlike fungal and bacterial co-infections in COVID-19 patients, comparatively HZ co-infection may not be a burden on mortality and morbidity. However, this question can be more accurately answered through a prospective study.

The principal strength of our review is the provision of a considerable amount of data on HZ virus co-infection in SARS-CoV-2-infected patients. This report opens door to further studies on this topic, especially prospective study designs, to validate our results. However, our review has some limitations that are inherent to the study design adopted. First, not all clinical cases were reported in the literature, and several SARS-CoV-2 disease and HZ co-infection cases (milder or more severe) could have existed and may have produced an impact on the findings of this pooled data. Second, we could not add any prospective studies in our review as none was found to be eligible. Third, the studies that we did add mainly included case reports. Fourth, we could not add the missed data and only the available information was collected. Moreover, Rash related to VZV reactivation could be asymptomatic or too mild to be noticed by clinicians, which led to selection bias whereby severe cases were more likely to be reported. Furthermore, patients may have had VZV-related rash and COVID-19 within a close time period solely through coincidence. Hence, it was difficult to differentiate whether rashes were produced from either COVID-19 or VZV reactivation, although dermatomal involvement was more in line with VZV reactivation. As this was a systematic review of case reports, case series, and retrospective studies, it could not establish a strong association between SARS-CoV-2 and HZV co-infection. However, this review does solidify the existence of the two together and invites further research on the impact of co-infection amid an ongoing pandemic.

## Conclusions

The recent SARS-CoV-2 pandemic has contributed to increased HZV reactivation, especially in elderly persons. Most cases developed HZ virus simultaneously or a few days after SARS-CoV-2 diagnosis, albeit some cases presented early during the prodromal period of SARS-CoV-2. Both psychological stresses during the pandemic and lymphopenia caused by SARS-CoV-2 led to immunosuppression that could be exploited by the HZ virus to reactivate and infect. Further research is warranted to establish the risk factors for HZ infection in SARS-CoV-2-infected patients, with a focus on the clinical outcomes.

## Declarations

### Ethics approval and consent to participate

Private information from individuals will not be published. This systematic review also does not involve endangering participant rights. Ethical approval is not required for this systematic review as only a secondary analysis of data already available in the electronic databases is conducted.

### Consent for publication

Written consent for the case report was obtained from the patient before submission of this manuscript.

### Availability of data and materials

Data sharing is not applicable.

### Competing interests

The authors declare that they have no competing interests.

### Funding

This study was not funded.

### Authors’ contributions

Conceptualization: Fateen Ata, Pawan Kumar Thada

Data curation: Muhammad Ali, Mohammad Nasser Affas, Jenish Bhandari, Sarosh Sarwar, Bilal Ahmed

Formal analysis: Mohammad Nasser Affas

Funding acquisition: Fateen Ata

Investigation: Fateen Ata, Pawan Kumar Thada, Muhammad Ali

Methodology: Fateen Ata

Project administration: Fateen Ata

Supervision: Fateen Ata

Literature review: Fateen Ata, Pawan Kumar Thada, Muhammad Ali, Mohammad Nasser Affas, Jenish Bhandari, Sarosh Sarwar, Bilal Ahmed

Writing - original draft: Fateen Ata, Pawan Kumar Thada, Muhammad Ali

Writing - review & editing: Fateen Ata

All authors read and approved the final manuscript.

### Acknowledgments

None.

## Figures and Tables

**Table 1:** Summary of the patients with herpes zoster and COVID-19

Characteristics		Results (n = 12)

Mean Age (Years)		Mean: 53.97 ± 18.91

Age	< 20 years	3 (3.8%)

groups	20–44 years	15 (19%)

	45–54 years	15 (19%)

	55–64 years	20 (25.31%)

	65–74 years	18 (22.78%)

	>75 years	8 (10.1%)

Gender		Males: 46 (58.2%) Females: 33 (41.8%)

Clinical	fever	39 (49.4%)

features	cough	27 (34.2%)

	dyspnea	13 (16.5%)

	myalgias	13 (16.5%)

	Loss of taste, smell	12 (15.2%)

	Sore throat	10 (12.7%)

	fatigue	4 (5.1%)

	diarrhea	4 (5.1%)

	Nasal congestion	1 (1.3%)

	vomiting	1 (1.3%)

	Chest pain	1 (1.3%)

	Loss of consciousness	2 (2.5%)


**Table 2:** Summary of the reported comorbidities of the patients

Previous Medical History	Frequency	Percent

HTN	11	13.9%

History of Chicken Pox	9	11.4%

History of Herpes Zoster in the past	5	6.3%

Recurrent infections	4	5.1%

Diabetes Mellitus	4	5.1%

Immunodeficiency	3	3.8%

Chronic Kidney Disease	3	3.8%

Malignancy	1	1.3%

Lung Disease	1	1.3%


**Table 3:** Summary of the clinical course for symptoms development and diagnosis

Characteristics	N	Median	Mean	Std

Duration of Symptoms before admission (days)	29	5	9.52	10.33

Duration from herpes zoster to COVID-19 diagnosis (days)	15	0	2.72	4.25

Duration from first COVID symptoms to herpes zoster diagnosis (days)	43	7	15.49	16.99

Duration from COVID-19 diagnosis to herpes zoster diagnosis (days)	44	6.50	14.80	17.03

Hospital stays (days)	3	32	49	44.03


**Table 4:** Distribution of HZ rash and treatment of patients added to the review

Author	Age/sex	No. of patients	Location of rash	Treatment

GHOSH B et al	26 yr. /M	1	Thoracic -abdomen tbl11-T12 dermatome	Oral Acyclovir, supportive management

Desai et al	62 yr./F	1	T11, tbl12	IV Acyclovir, amitriptyline, pregabalin

Kondo et al	57 yr./M	1	Located on the right side of the forehead in the first division of the trigeminal nerve	NA

Gupta et al	75 yr./F	1	NA	Oral valganciclovir, valaciclovir

Xu et al	73 yr. /M	1	Shoulder and neck	IV Acyclovir and supportive management

Fernandez-Nieto et al	56 yr./M,52 yr. /M,63 yr./ M,56 yr./F,82 yr./F,72 yr./F,78 yr./F	7	Ophthalmic in 2/ NA in others	Valacyclovir in 3 cases and NA in others

Karimi et al	12 yr. /M	1	Trunk. face and limbs	Acyclovir, acetaminophen

Ferreira et al	39 yr. /M	1	Left orofacial	Acyclovir, pregabalin

Patel et al	83 yr./M	1	NA	IV Acyclovir

Maldonado et al	25 yr./F	1	Right lumbar and right hand and leg	paracetamol, calamine lotion

Mar Llamas-Velasco	79 yr. /F	1	Anterior posterior trunk	NA

Puri et al	83 yr./M	1	NA	IV Acyclovir

Goyal et al	50 yr./M,60 yr./M	2	Right shoulder and back, left trunk	Acyclovir, topical fusidic acid cream

Voisin et al	80 yr./F	1	Chonca and external auditory canal	NA

Solanki et al	64 yr./M	1	Face bilateral, upper extremity	IV Valacyclovir

Katz et al	10 F,6 M	16	NA	NA

Elsaie et al	44 yr. /M	1	Front chest	Valacyclovir

Cao et al	70 yr./F	1	Right tbl10-T12	IV Acyclovir, pregabalin, ibuprofen

Shors et al	49 yr./F	1	FACE trigeminal V2	Valacyclovir, gabapentin. Topical lidocaine

Pona et al	70 yr. /F	1	left hip	Gabapentin

Saati et al	54 yr./M	1	right chest and tip of the scapula	Famciclovir, acetaminophen

Altaf et al	52 yr,75 yr,34 yr./all male	3	RIGHT SIDE OF NECK AND UPPER CHEST, right side of the chest, right tbl8	Oral acyclovir, prednisolone, calamine

Veraldi et al	48 yr./M	1	bilateral asymmetric dermatome	Valaciclovir, citalopram


**Table 5:** Summary of commonly reported medications used for COVID-19 and herpes zoster

Medications	Frequency	Percent

For COVID-19		

Hydroxychloroquine/chloroquine	14	17.8%

Antibiotics	8	10.1%

Remdesivir	3	3.8%

Oseltamivir	3	3.8%

AC (Enoxaparin/Heparin)	3	3.8%

Steroids	2	2.5%

Favipiravir	2	2.5%

Tocilizumab	2	2.5%

Ritonavir/Lopinavir	1	1.3%

For Herpes Zoster		

Acyclovir	42	53.2%

Valacyclovir	15	18.98%

Steroids (Dexamethasone, Prednisolone, Methylprednisolone)	11	13.9%

Calamine lotion	5	6.32%

Famciclovir	2	2.5%


**Table 6:** Patient's laboratory investigations in the early (first week) and late disease (second week) periods

Parameters	Early Disease (Day 1)	Late Disease (Day 14)

Hemoglobin (mg/dl)	10.1	10.6

Leukocyte Count (per mm3)	11650	13900

Lymphocytes	19%	4%

Platelets (per mm3)	195000	181000

D-Dimer (ng/ml)	783	1399

Ferritin (ng/ml))	398	12

CRP (mg/dl)	48	22

Lactate Dehydrogenase (u/l)	758	620

